# Acute Decompensated Heart Failure in the Elderly: An Observational Study in a Regional Victorian Hospital

**DOI:** 10.7759/cureus.51999

**Published:** 2024-01-10

**Authors:** Ali Uthuman, Tae H Kim, Josephine Wanandi, Ethan K Chan, Rachael S Lim

**Affiliations:** 1 Rural Health, University of Melbourne, Shepparton, AUS; 2 General Medicine, Goulburn Valley Health, Shepparton, AUS

**Keywords:** elderly population, regional australia, victoria, heart failure with preserved ejection fraction, heart failure with reduced ejection fraction

## Abstract

Objective

This study aimed to evaluate the frequency, triggers, clinical management, and outcomes of acute decompensated heart failure (ADHF) episodes in the elderly population of a regional Victorian town, along with analysing long-term outcomes, including rehospitalization rates, functional status, and mortality.

Methods

In this single-centre retrospective study, approved by the Research Governance Unit of Goulburn Valley Health, we analysed data from patients over 65 years of age discharged with a primary diagnosis of heart failure (HF) between July 2022 and June 2023. The study included 174 episodes from 148 patients, examining demographic and clinical profiles, investigations, outcome measures, and hospital admission risk program (HARP) involvement.

Results

The study highlighted a high prevalence of heart failure with preserved ejection fraction (HFpEF), especially in patients over 85 years. No significant association between sex and ejection fraction categories was observed. The average length of stay was 5.9 days, with longer stays noted for females. Non-invasive ventilation emerged as a significant predictor of extended hospitalization. A 30-day readmission rate of 6.67% was noted, lower than some existing studies.

Conclusion

The findings underscore the complexity of ADHF management in the elderly, suggesting the need for region-specific, gender-focused strategies and indicating the potential benefits of enhanced HARP program engagement. These insights contribute to a nuanced understanding of HF management in elderly populations in regional settings.

## Introduction

The prevalence of heart failure (HF) (of any ejection fraction (EF)) in Australia is between 1 and 2%, with an incidence rate of approximately 2.1 cases per 1000 individuals. Notably, HF tends to be more common among women and is more prevalent in rural and remote areas compared to metropolitan and capital regions [1].

In the context of acute decompensated heart failure (ADHF), studies have demonstrated high post-discharge mortality rates [2]. Furthermore, clinical outcomes for elderly ADHF patients may be comparably unfavourable when set against those of younger patients [3].

In response to the growing burden of HF, healthcare systems, particularly in Victoria, have implemented various support services aimed at facilitating self-management of the condition. A notable example is the hospital admission risk program (HARP), which has played a crucial role in providing necessary support to HF patients [4].

Our study aims to delve deeper into this issue, focusing specifically on elderly patients admitted with ADHF at a regional hospital in Victoria. The research is geared toward uncovering the triggers and outcomes and other critical epidemiological aspects of ADHF in this demographic to understand the disease's impact better and guide future healthcare strategies.

## Materials and methods

Ethical approval and design

Approval from the Research Governance Unit of Goulburn Valley Health (GVH) was granted for the single-centre retrospective study.

Inclusion criteria and data collection

Data were collected retrospectively for all patients above 65 who were discharged with a primary diagnosis of HF from 1st July 2022 to 30th June 2023. Coding results from the GVH database identified 174 episodes of admissions from 148 patients within this timeframe, which were then manually investigated to extract data related to the demographic profile (age, gender), the clinical profile (trigger, comorbidities), relevant investigations (left ventricular ejection fraction from a transthoracic echocardiogram done within twelve months of index admission, brain natriuretic peptide (BNP)), and outcome measures (non-invasive ventilator (NIV) use, length of stay (LOS), discharge destination, readmission), as well as the HARP involvement.

LVEF of above >50% was considered as heart failure with preserved ejection fraction (HFpEF), while 40 to 49% was taken as heart failure with mid-range ejection fraction (HFmrEF), and <40% was taken as HFrEF.

Hospital discharges without a primary diagnosis of HF and any patients with a primary diagnosis of HF who are less than 65 years of age were excluded.

Readmission criteria

We defined readmission as any hospital readmission related to ADHF within 30 days of a previous discharge, a standard benchmark in healthcare quality assessment. The 30-day criterion aligns with Australian healthcare standards. Readmissions within this timeframe often indicate ongoing health issues or challenges in care transition (avoidable hospital admissions) [5]. Admissions beyond the 30-day mark were treated as separate events or patients for statistical analysis, as they are less likely to be directly related to the initial hospitalisation.

Data processing and statistical analysis

Data were analysed using IBM SPSS Statistics for Windows, Version 29 (Released 2023; IBM Corp., Armonk, New York, United States). Descriptive statistical analysis presented summary statistics as mean with standard deviation and range. Where appropriate, inferential statistical tests were used to identify differences between groups. The chi-square test was used to compare categorical data. An independent samples t-test, the Mann-Whitney U test, ANOVA, and Dunn’s multiple comparison tests were used to compare categorical data to numerical data. Logistic regression analysis was used to investigate the odds of outcome measures between different variables. A p-value < 0.05 indicated statistical significance.

## Results

There was a total of 174 episodes involving 148 patients. The average age of participants was 83.18 years, with an age range from 65 to 99 years. In terms of sex distribution, there were 66 female (44.6%) and 82 male (55.4%) participants. Fifty percent of the patients were above the age of 85.

Regarding the involvement with the HARP, most participants, 68.9% (102 individuals), were not engaged with the program, whereas 31.1% (46 individuals) were part of the HARP initiative.

**Figure 1:**
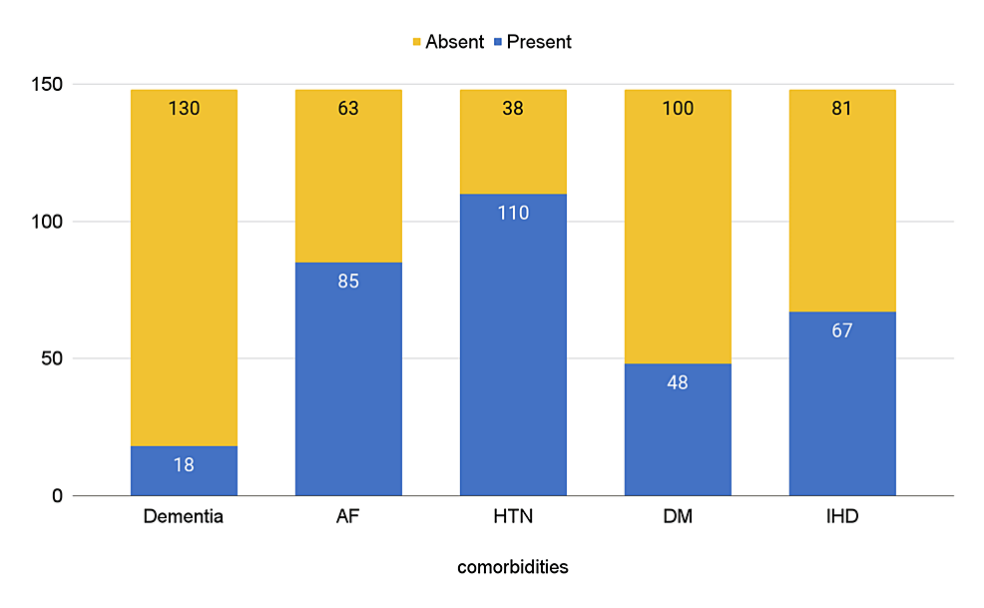
Prevalence of comorbidities among individuals AF: Atrial fibrillation, HTN: hypertension, DM: diabetes mellitus, IHD: ischaemic heart disease

**Figure 2:**
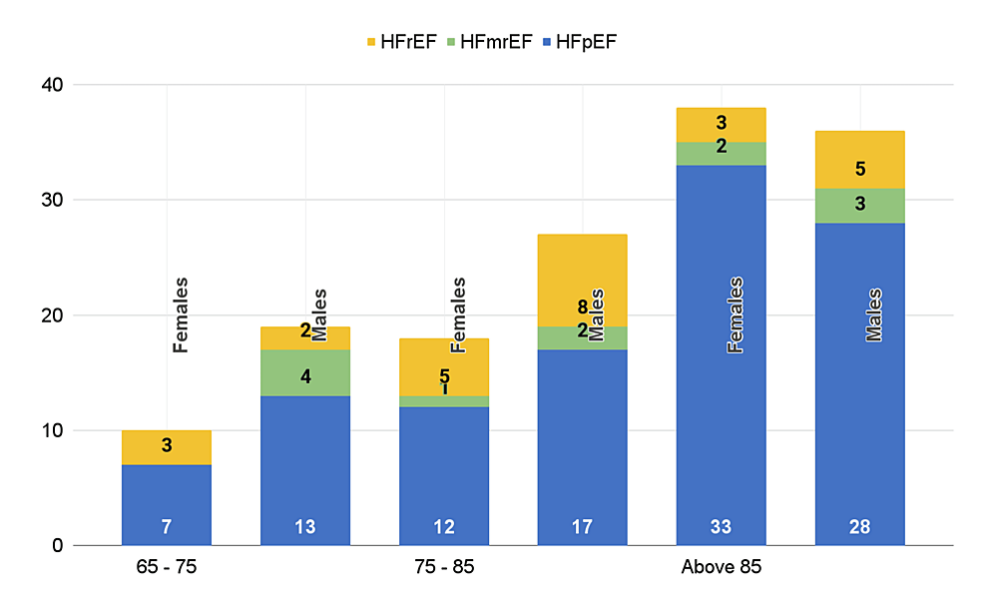
Relationship between the age group, EF, and sex HFrEF: Heart failure with reduced ejection fraction, HFmrEF: heart failure with mid-range ejection fraction, HFpEF: heart failure with preserved ejection fraction, EF: ejection fraction

The prevalence of common comorbidities among the participants is given in Figure 1. Figure 2 illustrates the relationship between the age group, EF, and sex.

The distribution of participants across EF categories revealed that 74.3% had HFpEF, 8.1% were classified with HFmrEF, and the remaining 17.6% had HFrEF.

The average LOS for hospital episodes was 5.91 days, ranging from 0.10 to 97.41 days. The most common reasons for hospitalisation were lower respiratory tract infections (21.3%), arrhythmias (5.7%), and medication non-adherence (6.3%). Most patients (71.3%) returned home upon discharge, 12.1% were transferred to residential aged care facilities (RACFs), and 5.2% passed away.

BNP levels in the study exhibited a wide range among the 87 patients analysed, with a mean of 9590.43 pg/mL and a median of 4673.00 pg/mL. The values varied significantly, as indicated by a significant standard deviation of 17961.33 pg/mL, from 602 to 138507 pg/mL.

ANOVA revealed significant age differences across the EF categories (p = 0.036). However, subsequent pairwise comparisons using the Tukey honestly significant difference (HSD) test did not demonstrate statistically significant differences between any specific EF groups. The chi-square test showed no significant association between sex and EF categories (χ² = 2.239, df = 2, p = 0.326).

A chi-square test of independence revealed a marginally non-significant association between EF groups and the presence of Atrial Fibrillation (AF), with a Pearson Chi-Square value of 5.536 (df = 2, p = 0.063) and a Likelihood Ratio of 5.757 (p = 0.056). Notably, a significant linear trend was observed in the data (Linear-by-Linear Association: χ²(1) = 4.826, p = 0.028), indicating an increasing prevalence of AF with more reduced EF HF categories - from HFpEF (51.8% AF prevalence) to HFmrEF (75% AF prevalence) and HFrEF (73.1% AF prevalence).

A t-test revealed that patients known to HARP services are 4.208 years older than those not involved with HARP (t = -2.935, df = 146, p = 0.004). Sex was not associated with the involvement of HARP service among patients, as demonstrated by a Pearson chi-square test (p = 0.854). The correlation between age and LOS is weak and not statistically significant (Pearson correlation = 0.056, p = 0.467).

The analysis of hospital stays by sex revealed a significant difference, with males having a shorter stay than females. An independent samples t-test confirmed this finding with a p-value of 0.008.

In the multiple linear regression analysis (MLR) examining factors influencing the LOS, the model significantly predicted LOS (F (10, 163) = 1.952, p = .042). Among the predictors, NIV emerged as a significant factor, with its usage associated with an increase in LOS by an average of 3.366 days (B = 3.366, p = .037). Additionally, the “transfer to other hospitals” within the outcome variable significantly influenced LOS, with an increase of 3.274 days (B = 3.274, p = .013). Other variables, including age, sex, and precipitating factors, did not significantly impact LOS.

**Figure 3:**
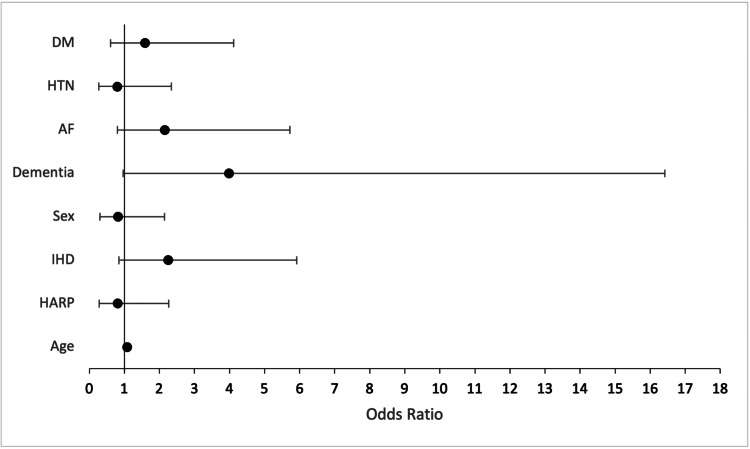
Coefficient plot of EF categories and predictors EF: Ejection fraction, DM: diabetes mellitus, HTN: hypertension, AF: atrial fibrillation, IHD: ischaemic heart disease, HARP: hospital admission risk program

In the multinomial logistic regression analysis to predict EF categories, the overall model did not statistically significantly differentiate between EF groups (p = 0.104). Despite this, individual predictors showed some notable effects. Age emerged as a significant predictor; with each additional year, the odds of being classified in the HFpEF category (compared to HFrEF) increased by 7.2% (Odds Ratio = 1.072, p = 0.023), when controlling for other variables. However, no other predictors, including sex, HARP involvement, dementia, AF, HTN, DM, and IHD, demonstrated significant effects in predicting EF categories (Figure 3).

In our subset analysis of BNP, we observed no significant correlations with age (Pearson correlation = 0.003, p = 0.975), sex (Mann-Whitney U = 1006.000, p = 0.406), or length of stay (correlation = 0.114, p = 0.292). Additionally, the Kruskal-Wallis test indicated no significant differences in BNP levels across EF categories (p = 0.955).

A total of 10 patients were readmitted, and their characteristics are detailed in Table 1. All ten patients exhibited AF and HTN. Six of them were aged above 85 years, and 70% were diagnosed with HFpEF. Additionally, six of these readmitted patients were subsequently discharged to RACFs. Notably, only two of them had prior involvement with the HARP.

**Table 1:** Characteristics of patients with readmission M: Male, F: female, LRTI: lower respiratory tract infection, NH: nursing home, Rehab: rehabilitation ward, HFpEF: heart failure with preserved ejection fraction, HFmrEF: heart failure with mid-range ejection fraction, HFrEF: heart failure with reduced ejection fraction, AF: atrial fibrillation, HTN: hypertension, DM: diabetes mellitus, IHD: ischaemic heart disease

Age	Sex	Trigger	HARP	Discharge	EF	AF	HTN	DM	IHD
74	M	Other	No	Demise	HFpEF	Yes	Yes	No	Yes
84	F	LRTI	Yes	NH	HFpEF	Yes	Yes	No	Yes
95	F	Other	No	NH	HFpEF	Yes	Yes	Yes	Yes
86	F	Other	No	Rehab	HFpEF	Yes	Yes	Yes	Yes
94	M	Med	No	NH	HFrEF	Yes	Yes	No	No
81	F	Other	No	NH	HFpEF	Yes	Yes	Yes	Yes
89	M	Other	No	NH	HFpEF	Yes	Yes	Yes	Yes
89	M	Other	Yes	Rehab	HFrEF	Yes	Yes	Yes	Yes
66	M	LRTI	No	Home	HFpEF	Yes	Yes	No	No
92	F	LRTI	No	NH	HFrEF	Yes	Yes	Yes	Yes

## Discussion

Our study revealed a substantial occurrence of HFpEF, particularly among patients older than 85, suggesting a trend towards higher EJs with advancing age. This observation aligns with the findings of Hyakuna et al. in Japan, where very elderly patients exhibited elevated EFs [6]. Similar results were reported by Shariff et al. [7]. Furthermore, the participation in the HARP program was 31%, surpassing the 20% reported in the disease management program by Parsons et al. [8].

Our study did not identify a significant association between sex and EF categories. However, this finding contrasts with the work of Kaur et al. and Tadic et al., both of whom reported a higher prevalence of HFpEF among women [9,10]. This discrepancy may suggest variations in patient populations or methodological approaches across studies.

The overall association between AF and EF categories did not reach statistical significance in our investigation. However, a notable linear-by-linear association was observed, particularly between AF with HFmrEF and HFrEF. This trend aligns with findings from a 2022 Japanese study, which reported that even a mild reduction in EF at enrolment was linked to increased hospitalisations in patients with AF [11]. These results underscore the potential impact of even minor EF impairments in the AF patient population, suggesting a need for heightened vigilance and targeted management strategies in this subgroup.

Our study reported an average LOS of 5.9 days, which falls within the range observed by Al-Omary et al. in their 2018 Australian study, where LOS varied between 5 and 8 days. Notably, our findings are in close agreement with the LOS of 6.4 days reported for patients in rural areas in the Al-Omary et al. study [12].

While our t-test results indicated a statistically significant longer LOS for females compared to males, this association was not corroborated by our multiple linear regression analysis. These findings are partially consistent with those of Tigabe et al. in Ethiopia in 2021, who reported an increased LOS among female patients [13]. In contrast, the Aurora Study in 2020 demonstrated that being male was associated with increased LOS [14]. This discrepancy highlights the complexity of factors influencing LOS and suggests that a multifactorial approach, rather than a focus on a single variable like gender, is necessary to understand and manage LOS in ADHF patients effectively.

Moreover, the regression suggested that using NIV significantly predicts extended hospitalisation. Miller et al. also identified ventilation contributing to longer hospital stays [15]. It is apparent that patients who need ventilatory support are critically ill and need a longer hospital stay.

In our cohort, the specific triggers for ADHF were predominantly unidentified; however, we did identify that 21% of the episodes were associated with lower respiratory tract infections, arrhythmias accounted for about 6%, and medication non-adherence was responsible for 6.5% of the cases. This contrasts with findings from the study by Newton et al. in NSW in 2016, where atrial fibrillation/flutter was present in 42% of patients, though only deemed the primary cause in 15%, a considerably higher rate than our study [16]. Furthermore, a study from Melbourne in 2019 cited infections, improper IV fluid administration, and tachyarrhythmias as common precipitants of ADHF [17].

In our study, the ANOVA indicated significant age differences across EF categories (p = 0.036), suggesting variability in age distribution among different EF groups. However, the lack of statistically significant differences in the Tukey HSD post hoc comparisons implies that these age differences are not distinctly pronounced between any specific EF groups. This finding is intriguingly contrasted by our multinomial logistic regression analysis, where age was a significant predictor in distinguishing between HFpEF and HFrEF categories (Odds Ratio = 1.072, p = 0.023). This discrepancy suggests that while age plays a role in differentiating between the broad categories of HFpEF and HFrEF, it does not distinctly demarcate the subcategories within this spectrum. This nuanced understanding underlines the complexity of age as a determinant in heart failure categorization and highlights the need for further investigation into other contributing factors that delineate these EF categories.

Our analysis found no statistically significant associations between BNP levels, sex, and EF categories. This contrasts with findings by Hsich et al., who reported higher BNP levels in HFrEF compared to HFpEF and lower levels in females [18]. A possible explanation for this discrepancy in our study might be the inclusion of HFmrEF as a distinct group and the relatively small sample size, which could have impacted the detectability of differences in BNP levels across these categories.

In our analysis, the 30-day readmission rate for patients was found to be 6.67%, which is notably lower than the 20% rate reported in a 2018 Australian study [19]. This difference underscores potential variations in patient demographics, healthcare systems, or discharge and follow-up protocols between the two study settings. Additionally, 60% of readmitted patients did not have an overt trigger. Moreover, it is unclear whether having AF would have had any contribution to the admissions as a trigger.

Our observation that 60% of readmitted patients were discharged to RACFs could imply a significant functional decline during hospitalisation for ADHF. This aligns with findings from Yaku in 2020, which indicated that 15% of ADHF patients experienced a functional decline, particularly among those over 80 years old, female patients, and other risk groups [20]. This pattern highlights the potential impact of ADHF hospital stays on patient mobility and independence.

Limitations

This study, while providing valuable insights into ADHF among the elderly population in a regional Victorian town, has several limitations. Firstly, the study's observational nature limits our ability to establish causal relationships between HF characteristics and patient outcomes. Additionally, the study's focus on a specific regional area may limit the generalizability of the findings to other geographical locations or populations with different socio-economic backgrounds.

The small sample size, especially in subgroup analyses (such as those involving the HARP program and specific EF categories), may have reduced the statistical power to detect significant differences or associations. Furthermore, the lack of longitudinal follow-up restricts our understanding of the long-term outcomes and progression of HF in this population. The study also did not account for certain variables, such as socioeconomic status and specific lifestyle factors, which could influence HF management and patient outcomes. Lastly, the reliance on hospital records and patient recall for data collection might have introduced information bias, particularly in aspects like medication adherence and the precise identification of triggers for HF exacerbation.

## Conclusions

Our study provides valuable insights into managing ADHF in older people of a regional Victorian town, highlighting the predominance of HFpEF in those over 85. This finding, mirroring global trends, underscores the complexities of managing heart failure in an aging demographic and stresses the need for tailored clinical strategies in geriatric care. Additionally, the lack of a significant association between sex and EF categories challenges existing beliefs and points to the role of regional and demographic factors in heart failure presentations. The longer average LOS in the hospital for females and the impact of NIV on LOS further indicate the necessity of gender-specific and condition-specific approaches in patient management.

The study also highlights the underutilised but impactful HARP, suggesting opportunities for better patient engagement in disease management. Notably, the common triggers for ADHF, such as LRTI and arrhythmias, emphasise the need for vigilant monitoring and preventive strategies in this cohort. Despite its regional focus and limited sample size, our study adds to the understanding of heart failure in elderly populations, particularly in regional contexts, underlining the importance of ongoing research to refine patient care, inform clinical practice, and shape health policy.
